# Non-Targeted Metabolomics and Network Pharmacology Reveal Bioactive Metabolites and the Medicinal Potential of Three Ornamental Camellia Flowers

**DOI:** 10.3390/plants14192967

**Published:** 2025-09-24

**Authors:** Yali Zhang, Jianhua Zhang, Yani Wu, Yin Wu, Wenjiao Guo, Chunshan You

**Affiliations:** 1 Forestry and Fruit Research Institute, Shanghai Academy of Agricultural Sciences, Shanghai 201403, China; 2 N.O.D Topia (Guangzhou) Biotechnology Co., Ltd., Guangzhou 510000, China; 3 Guangdong Sensitive Skin Care Engineering Technology Research Center, 544 Guangzhou Avenue North, Guangzhou 510000, China; 4 School of Design, Shanghai Jiao Tong University, Shanghai 200240, China

**Keywords:** *Camellia*, non-targeted metabolomics technology, network pharmacology, phenylpropanoids and polyketides, inflammation, oxidative stress, antibacterial, antiviral

## Abstract

The genus *Camellia* offers valuable resources for tea production, oil extraction, and ornamental purposes, and its applications are expanding beyond traditional regions due to increasing human demands and advancements in research. To explore new therapeutic resources and identify key active metabolites, we conducted a non-targeted metabolomics analysis on three camellias. We also utilized network pharmacology to identify the potential targets of key metabolites involved in anti-inflammatory, antioxidant, antibacterial, and antiviral effects. A total of 385 significantly different metabolites were identified, with organic acids and derivatives, lipids and lipid-like molecules, and phenylpropanoids and polyketides being the top three metabolite classes. Of the 71 different phenylpropanoids and polyketides identified, 54 were common across all three cultivars, while 17 were unique. Network pharmacology further identified 78 potential molecular targets associated with the four therapeutic activities under study. Seven flavonoid glycosides, two flavans, two biflavonoids/polyflavonoids, and one flavone were highlighted as key active metabolites. Notably, *Camellia japonica* ‘Kōshi’ emerged as a promising material for future applications. The key active ingredients may contribute to the development of novel approaches for cosmetic, food, and medicinal applications, as well as germplasm innovation for new functional camellias.

## 1. Introduction

The genus *Camellia* is an important economic and ornamental resource, with *Camellia sinensis* cultivated for tea, *Camellia oleifera* for oil production, and *Camellia japonica* for ornamental purposes. Recently, several sections of the genus, including section *Chrysantha*, section *Camellia*, section *Thea*, and section *Paracamellia*, have attracted increasing scientific interest due to their abundance of bioactive compounds and potential applications in food, cosmetic, and medicinal industries [1,2,3]. Among these, plants in section *Camellia* and section *Chrysantha* are valued not only as ornamental plants but also for their therapeutic potential and frequent use as cosmetic ingredients [4,5]. The oil extracted from *C. japonica* has been identified as a potential anti-asthmatic agent, with oleic acid recognized as its principal bioactive component [6]. Furthermore, this oil has been proposed as a natural preservative in the pharmaceutical industry [7], while its flowers are considered edible or suitable for beverages [8,9]. Within section *Chrysantha*, *Camellia nitidissima* is one of the most extensively studied species for medicinal applications [10], although several other species are also under investigation [11].

Metabolites profiling of camellias has revealed considerable chemical diversity. Using widely targeted metabolomics, 131 metabolites were identified from the leaf extracts of six golden camellias, with flavonoids (41 compounds) and amino acids (32 compounds) dominating the metabolite profiles [11]. Similarly, a targeted metabolomics study of *Camellia tienii* flowers and leaves identified 126 metabolites, the majority of which were flavonoids [12]. In another study, flower extracts from eight *C. japonica* cultivars were subjected to untargeted metabolomics for polyphenol profiling. Notably, extracts from *C. japonica* ‘Carolyn Tuttle’ exhibited high flavone accumulation, which correlated with strong free radical–scavenging activity and pronounced cytotoxicity against the Caco-2 cell line [13]. Despite the existence of approximately 30,000 camellias, current research on their metabolites remains limited.

Network pharmacology, first introduced in 2007 by British pharmacologist Andrew L. Hopkins, has emerged as a valuable tool in drug discovery and mechanistic studies. This approach integrates systems biology, bioinformatics, and computational network science to systematically analyze the complex molecular interactions between drugs and the human body, thereby providing insights into pharmacological mechanisms [14,15,16]. Applications of network pharmacology in camellia research include evaluating the anti-inflammatory activity of black tea from *Camellia ptilophylla* [17], investigating the mechanisms by which camellia species may alleviate osteoporosis [18], predicting the potential molecular mechanisms of *C. nitidissima* in the treatment of lung cancer [2], and analyzing the role of *C. sinensis* in the treatment of hypertension, cancer, and inflammation [19].

Flavonoids are bioactive compounds found in plants, and they are attracting increasing attention as functional foods or medicinal agents for various diseases [20,21,22]. Their roles in cancer [23,24], Alzheimer’s disease [25], diabetes [26], age-related neurodegenerative disorders [27], and other diseases have been explored. Within the genus *Camellia*, flavonoids have been studied for their antitumor, antifungal, anti-osteoporotic, and cardioprotective activities [28,29]. For example, total flavones have shown protective effects against cerebral ischemic injury and myocardial ischemia [30,31], while the anticancer activity and mechanisms of *C. nitidissima* have been documented [2]. Flavonoids from *C. oleifera* leaves exhibit significant inhibitory activity against certain food spoilage and pathogenic bacteria [32], and the strong antioxidant properties of tea are largely attributed to its flavonoid content [33]. Collectively, these findings underscore the remarkable potential of camellias not only as ornamental plants but also as economically valuable sources of bioactive compounds, particularly flavonoids.

Building on existing research into the metabolites and applications of camellias, and considering recent technological advances, it is evident that camellia-based functional studies offer substantial opportunities for future development. To further investigate this potential, we adopted a dual-pronged strategy to examine metabolic differences among three ornamental camellias and predict their potential therapeutic functions. First, non-targeted metabolomics was used to systematically characterize metabolites across different cultivars, with particular emphasis on flavonoids, especially those derived from phenylpropanoid and polyketide pathways. Second, network pharmacology analysis was conducted to predict key active ingredients associated with anti-inflammatory, antioxidant, antibacterial, and antiviral activities. This integrative approach advances both the phytochemical understanding of camellia flowers and provides a methodological framework for targeted breeding and accelerates the discovery of functional, plant-derived compounds within the genus.

## 2. Results

### 2.1. Overview of Metabolomics Analysis

#### 2.1.1. Multivariate Statistical Analysis of Three Camellias

**Figure 1:**
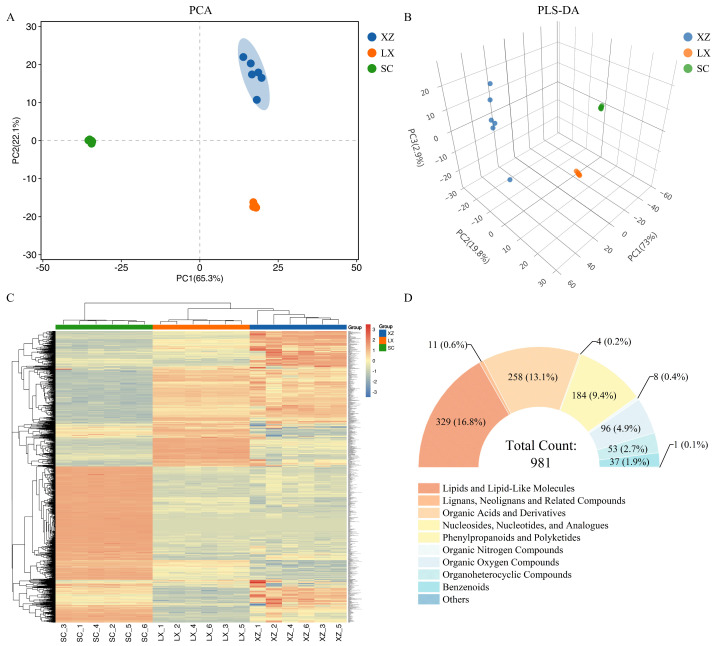
Multivariate statistical analysis of three camellias. (**A**) PCA of the identified metabolites. (**B**) Three-dimensional PLS-DA score plot showing metabolic differentiation. (**C**) Heat map of metabolites, with samples displayed horizontally and metabolites vertically. (**D**) Classification of metabolites at the super class level, showing the 10 categories and their count percent.

Principal component analysis (PCA) revealed clear differences in metabolic phenotypes among the three camellias studied. The first principal component (PC1) accounted for 65.3% of the total variance, while the second principal component (PC2) explained 22.1%. Samples clustered tightly within each group but were distinctly separated along the two axes (Figure 1A), indicating pronounced metabolic divergence among the cultivars. Partial least squares–discriminant analysis (PLS-DA) further confirmed this separation. In the three-dimensional PLS-DA score plot (Figure 1B), the cultivars were well discriminated from one another. The cumulative R^2^Y and Q^2^ values were 0.999 and 0.995, respectively, demonstrating both the robustness and predictive reliability of the model. All metabolites from the three cultivars fell within the confidence intervals (Figure S1), supporting the stability of the PLS-DA model.

Hierarchical clustering analysis visualized in a heat map (Figure 1C) also grouped the metabolites into three distinct clusters, consistent with cultivar-specific metabolic profiles. In total, 981 metabolites were identified and annotated across all samples, and these were categorized into 10 groups (Figure 1D): lipids and lipid-like molecules (33.54%), organic acids and derivatives (26.30%), phenylpropanoids and polyketides (18.76%), organic oxygen compounds (9.79%), organoheterocyclic compounds (5.40%), benzenoids (3.77%), lignans, neolignans, and related compounds (1.12%), organic nitrogen compounds (0.82%), nucleosides, nucleotides, and analogs (0.41%), and others (0.10%). Notably, lipids and lipid-like molecules were the most abundant category, followed by organic acids and derivatives, and phenylpropanoids/polyketides.

#### 2.1.2. Screening of Different Metabolites Among Three Camellias

**Figure 2:**
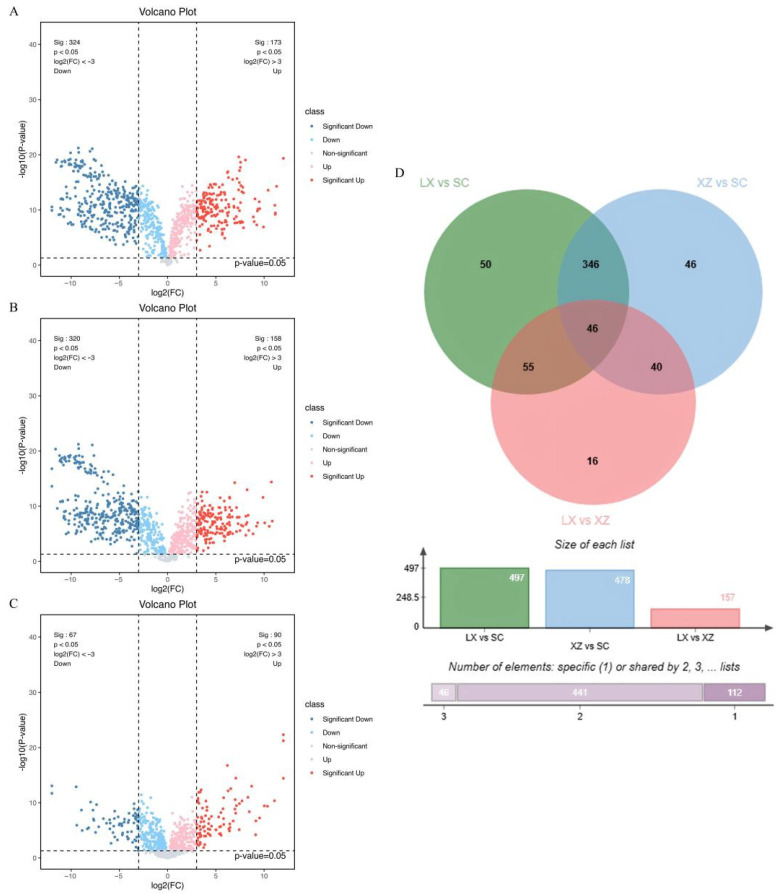
Analysis of metabolite differences among three camellias. (**A**) Volcano plot showing metabolites with higher and lower abundance in LX compared to SC. (**B**) Volcano plot showing metabolites with higher and lower abundance in XZ compared to SC. (**C**) Volcano plot showing metabolites with higher and lower abundance in LX compared to XZ. (**D**) Venn diagram showing the overlap of differentially accumulated metabolites among the comparison groups.

Among the 981 identified metabolites, 949 were significantly different across the three camellias with fold change (FC) ≥ 8 or ≤1/8 and *p* values < 0.05. Pairwise comparisons revealed 497 differential metabolites between *C. japonica* ‘Kōshi’ (XZ) and *C. japonica* (SC), 478 between *C.* ‘High Fragrance’ (LX) and SC, and 157 between LX and XZ. These results indicate that LX and XZ are more metabolically similar to each other than either is to SC. In the LX vs. SC comparison (Figure 2A), 497 differential metabolites were detected, of which 173 were more abundant and 324 less abundant in the LX group compared to the SC group. The XZ vs. SC comparison (Figure 2B) yielded 478 differential metabolites, including 158 more abundant and 320 less abundant in the XZ group compared to the SC group. By contrast, the LX vs. XZ comparison (Figure 2C) showed only 157 differential metabolites, with 90 more abundant and 67 less abundant in the LX group compared to XZ. These metabolite distribution patterns highlight distinct chemical differences among the three cultivars and provide a basis for identifying compounds of potential biological or economic value.

To further refine the analysis, metabolites with a variable importance in projection (VIP) value > 1 from the Orthogonal Partial Least-Squares–Discriminant Analysis (OPLS-DA) model were combined with differential metabolites identified from volcano plots. A Venn diagram (Figure 2D) illustrated that LX and XZ shared the greatest similarity, with only 16 unique differential metabolites. In comparison, LX vs. SC and XZ vs. SC contained 50 and 46 unique differential metabolites, respectively. These metabolites may play important roles in studying the genetic differences or diversity of the three camellias. Notably, 46 metabolites were consistently different across all three pairwise comparisons, suggesting that these common metabolites may represent core metabolic features with fundamental roles across the cultivars.

### 2.2. Deep Analysis of Significantly Different Phenylpropanoids and Polyketides in Three Camellias

#### 2.2.1. In-Depth Examination of Differential Metabolites

**Figure 3:**
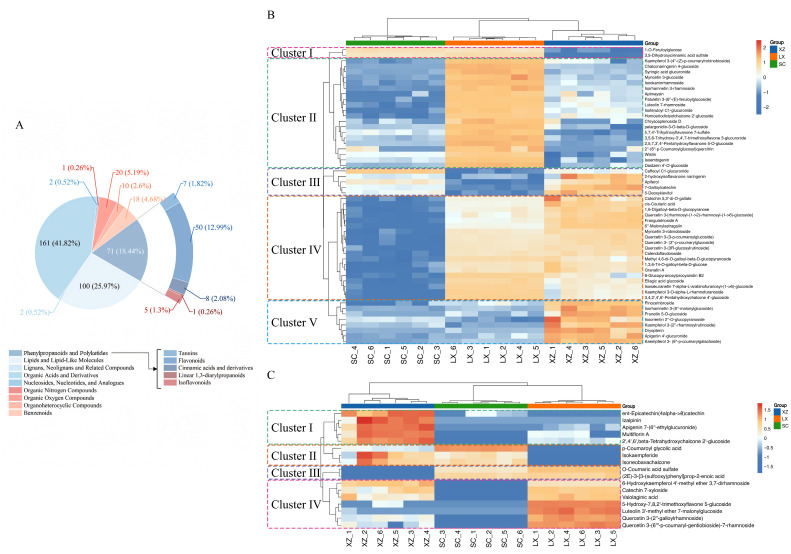
Analysis of differentially abundant metabolites. (**A**) Superclass- and class-level classification of differential metabolites. (**B**) Heat map of common phenylpropanoids and polyketides based on hierarchical clustering. (**C**) Heat map of unique phenylpropanoids and polyketides across three camellias based on hierarchical clustering.

From the 949 significantly different metabolites, 385 were retained for detailed analysis based on the criteria VIP > 1, *p* < 0.05, and fold change (FC) ≥ 8 or ≤1/8. These metabolites were classified at both the superclass and class levels to highlight variations in phenylpropanoid and polyketide compounds, particularly flavonoids. The selected 385 metabolites were classified into nine groups (Figure 3A), with the three most abundant being organic acids and derivatives (161), lipids and lipid-like molecules (100), and phenylpropanoids/polyketides (71). Within the phenylpropanoid and polyketide group, flavonoids accounted for 50 compounds, representing 70.4% of this category.

#### 2.2.2. Differential Phenylpropanoids and Polyketides Analysis Among the Three Camellias

To further characterize metabolic variation, 71 phenylpropanoids and polyketides were further analyzed. Metabolites absent in all six replicates or undetected in more than half of the replicates for one or two cultivars were classified as unique. Based on this criterion, 17 phenylpropanoids and polyketides were identified as unique, while the remaining 54 phenylpropanoids and polyketides were common to all three camellias.

Despite being shared, the relative abundances of these 54 metabolites varied considerably and could be grouped into five distinct clusters (Figure 3B). Cluster I comprised two cinnamic acid derivatives that showed the lowest accumulation in XZ. Cluster II contained 20 metabolites, including 15 flavonoids, which accumulated most strongly in LX. Cluster III consisted of one cinnamic acid derivative, two isoflavonoids, and two flavonoids, with LX exhibiting the lowest levels in this group. Cluster IV included 19 metabolites, largely flavonoids, that accumulated least in SC; notably, all quercetin derivatives, key bioactive components in camellia, were present in this cluster. Finally, Cluster V comprised eight metabolites, six of which were flavonoids, with XZ showing the highest accumulation. Taken together, these results indicate that the relative abundance of phenylpropanoids and polyketides was consistently higher in XZ and LX compared with SC, particularly with respect to flavonoid content, highlighting their potential importance as sources of bioactive compounds.

Among the 17 unique metabolites depicted in Figure 3C, XZ displayed the highest number and relative abundance, whereas SC exhibited the lowest. Only two metabolites, O-coumaric acid sulfate and (2E)-3-[3-(sulfooxy)phenyl]prop-2-enoic acid, were absent in XZ, while nine metabolites were undetected in SC. Five metabolites, including p-coumaroyl glycolic acid, isokaempferide, isoneobavachalcone, izalpinin, and ent-epicatechin(4alpha->8) catechin, were undetectable in LX. In addition, izalpinin was exclusively detected in XZ and has been reported as an active ingredient of *Alpiniae oxyphyliae* Fructus with anti-Alzheimer’s disease properties [34]. The 17 unique metabolites were further classified into four clusters (Figure 3C). Metabolites in Clusters I and IV were predominantly flavonoids, with the lowest accumulation observed in SC, whereas metabolites in Clusters II and III accumulated most highly in SC. Overall, the distinct profiles of unique metabolites among the three camellias highlight potential genetic differences and underlying metabolic diversity.

### 2.3. Prediction of Potential Active Compounds and Targets via Network Pharmacology

#### 2.3.1. Active Compounds and Disease-Associated Targets of the Studied Camellias

**Figure 4:**
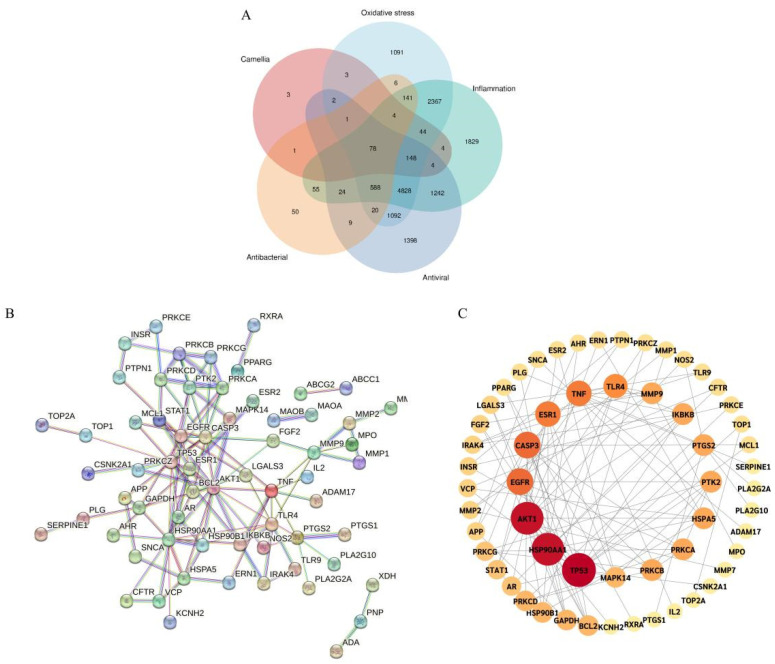
Intersection and core targets associated with the *Camellia* cultivars. (**A**) Venn diagram illustrates the overlapping targets associated with anti-inflammatory, antioxidant, antibacterial, and antiviral targets. (**B**) PPI network of the selected targets. (**C**) Schematic representation highlighting core targets within the network.

Using the 71 selected common and unique phenylpropanoid and polyketide metabolites from the three camellias, target prediction was performed with the Swiss Target Prediction platform, resulting in a total of 292 compound-associated targets. To identify disease-relevant targets, the Drug Bank database was searched for targets associated with anti-inflammatory, antioxidant, antibacterial, and antiviral activities, yielding 11,356, 10,413, 977, and 9434 targets, respectively. The intersection between these disease-associated targets and the predicted compound targets was visualized using a Venn diagram generated Via the Chinese Version of Bioinformatics (https://www.bioinformatics.com.cn/, (accessed on 20 May 2025)). This analysis identified 78 potential intersection targets between the camellia metabolites and the four disease categories, which were considered candidates for further functional investigation (Figure 4A).

#### 2.3.2. Construction of PPI and Selection of Core Targets

The 78 intersection targets (Table S1) were imported into the STRING database, with the species set to *Homo sapiens* and the confidence level adjusted to high (0.900) to construct a protein–protein interaction (PPI) network (Figure 4B). The resulting network consisted of 78 nodes and 121 edges, with an average node degree of 3.1 and a clustering coefficient of 0.441, illustrating the interactions among the intersection targets. In the network, each node represented a protein, and the edge length reflected the strength of interaction between the targets.

Network data were further analyzed in Cytoscape 3.10.2 to calculate degree centrality (DC). Nodes with higher DC values appear larger and darker in the network, indicating their critical importance. According to the PPI network analysis, the top five targets were identified as Tumor Protein 53 (TP53), Heat Shock Protein 90kDa Alpha (Cytosolic), Class A Member 1 (HSP90AA1), AKT serine/threonine kinase 1 (AKT1), Cysteine–Aspartic Acid Proteinase 3 (CASP3), and Epidermal Growth Factor Receptor (EGFR) (Figure 4C).

#### 2.3.3. GO Function and KEGG Pathway Enrichment Analysis

**Figure 5:**
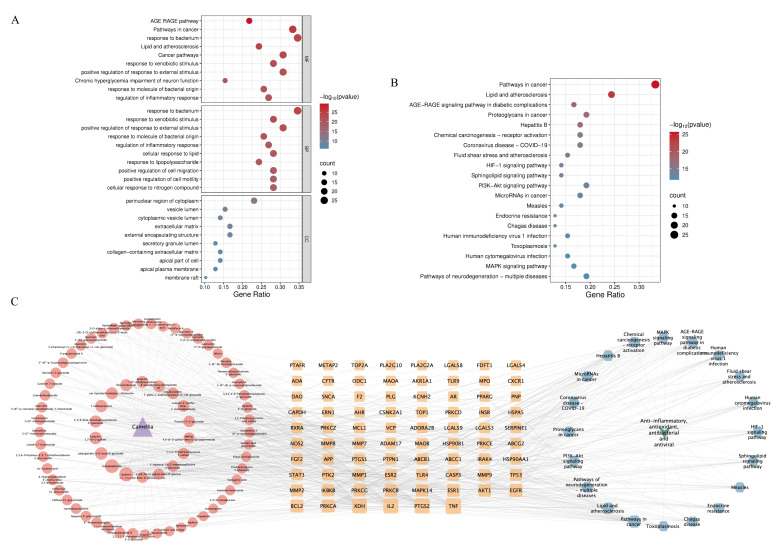
GO, KEGG, and network analyses in network pharmacology. (**A**) GO enrichment analysis of potential disease targets. (**B**) KEGG pathway enrichment analysis of the potential disease targets. (**C**) Network illustrating “Camellia-ingredient-intersection targets-pathway-disease”.

GO enrichment and KEGG pathway analyses were conducted using the 78 targets via the Metascape database. GO functional enrichment analysis (set at *p* < 0.01) identified 1177 biological processes (BP), 74 cellular components (CC), and 1780 molecular functions (MF). As shown in the GO bubble plots (Figure 5A), BP was primarily associated with responses to bacterium, xenobiotic stimulus, external stimulus, molecules of bacterial origin, and inflammation. MF was primarily related to the advanced glycation end product (AGE)-RAGE (receptor for AGEs) pathway, pathways in cancer, response to bacterium, lipid and atherosclerosis, and cancer pathways. CC mainly affected the perinuclear region of the cytoplasm, vesicle lumen, cytoplasmic vesicle lumen, extracellular matrix, and external encapsulating structure.

KEGG pathway enrichment analysis (*p* < 0.01) identified 172 relevant pathways (Figure 5B). These were mainly concentrated in cancer-related signaling pathways, lipid and atherosclerosis pathways, the AGE-RAGE signaling pathway in diabetic complications, proteoglycans in cancer, and hepatitis B. These results indicate that the phenylpropanoid and polyketide compounds from the three camellias may exert therapeutic effects through multiple interconnected biological pathways.

#### 2.3.4. Network Diagram Construction and Key Ingredients Identification

A comprehensive “Camellia-ingredient-intersection targets-pathway-diseases” network was constructed using Cytoscape 3.10.2. This network comprised 172 nodes and 754 edges. Red nodes represent intersecting targets; the orange nodes represent phenylpropanoids and polyketides in the three camellias; the blue nodes represent the top 20 signaling pathways; the green nodes represent the four functional categories (anti-inflammatory, antioxidant, antiviral, and antibacterial),; and the purple nodes denote the camellias. Larger nodes indicate higher degree values and a greater number of connected nodes.

Analysis of degree values identified the top 12 compounds as potential key active ingredients (Table 1). These included seven flavonoid glycosides, two flavans, two biflavonoids or polyflavonoids, and one flavone. Notably, all 12 compounds were present in XZ, whereas the other two cultivars lacked certain compounds. For instance, izalpinin, 2′,4′,6′-beta-tetrahydroxychalcone 2′-glucoside, apigenin 7-(6″-ethylglucuronide), and luteolin 3′-methyl ether 7-malonylglucoside were not detected in SC, whereas izalpinin and ent-epicatechin (4alpha->8) catechin were not detected in LX. Izalpinin, which was detected exclusively in XZ, exhibited the highest degree score, indicating its central role in the network. Isokaempferide demonstrated the highest accumulation across all three camellias, ranking second in degree value. These findings suggest that XZ may serve as a particularly rich source of bioactive flavonoids with potential multi-target therapeutic effects.

## 3. Discussion

### 3.1. Valuable Resources Improve the Application of Genus Camellia

Recent explorations of wild populations and advances in breeding have expanded the diversity of species and cultivars in the genus *Camellia*. This growing diversity offers valuable resources for research in tea production, medicinal applications, horticulture, and other areas of interest. Historically, the flowers of *C. japonica* and *C. nitidissima* have been the most commonly studied and utilized materials [16,35,36,37,38]. However, interest is increasingly shifting toward other camellia species or cultivars. For instance, previous studies on members of Sect. *Chrysantha* have highlighted the high-value potential of the golden camellia [11,12,13,38]. Building on this knowledge, future research may identify new camellia materials with even higher levels of bioactive compounds than *C. nitidissima*. In our study, two highly fragrant camellias (XZ and LX) were selected and compared with the commonly used SC. The results revealed significant metabolic differences: both XZ and LX contained higher relative levels of phenylpropanoids and polyketides than SC, and their key bioactive ingredients associated with four therapeutic functions were distinctive. These findings enhance our understanding of metabolic variation among camellias and highlight XZ and LX as potential resources for applications in cosmetics, food, medicine, and related fields.

### 3.2. More Flavonoid Components Are Identified as Vital Bioactivities

Anti-inflammatory, antioxidant, antibacterial, and antiviral properties are considered vital bioactivities in camellia research, with catechin and epigallocatechin gallate (EGCG) being among the most widely studied compounds [39,40]. Advances in metabolite profiling and network pharmacology have enabled researchers to focus on specific flavonoid components. For example, network pharmacology combined with experimental validation identified luteolin, kaempferol, quercetin, eriodictyol, and 3′4-O-dimethylcedrusin as key active ingredients of *C. nitidissima* against lung cancer [2]. In black tea from *C. ptilophylla*, luteolin, quercetin, anthocyanins, apigenin, and 10 additional polyphenols significantly inhibited nitric oxide production, as revealed by high-resolution mass spectrometry and network pharmacology analyses [17]. Similarly, 96 pharmacologically active metabolites were identified in *Camellia tuberculata* seeds, showing potential effects against tumors, cardiovascular disease, diabetes, atherosclerosis, and osteoporosis. Some metabolites were associated with multiple disease categories, including isorhamnetin-3-O-glucoside and quercetin (seven diseases), vitexin and dehydrodiisoeugenol (six diseases), nobiletin and fraxetin (five diseases), resveratrol (four diseases), and rutin (three diseases) [41]. Flavonoids F2 (kaempferol 3-O-[β-d-glucopyranosyl-(1→2)-α-l-rhamnopyranosyl-(1→6)]-β-d-glucopyranoside) and J2 (kaempferol 3-O-[β-d-xylopyranosyl-(1→2)-α-l-rhamnopyranosyl-(1→6)]-β-d-glucopyranoside), isolated from *C. oleifera* meal, both exhibited excellent antibacterial activity [42], while quercetin 3-O-β-d-galactopyranoside demonstrated potent 1,1-diphenyl-2-picrylhydrazyl (DPPH) radical-scavenging activity [43].

In the present study, quercetin, apigenin, epicatechin, and luteolin were among the metabolites detected. Among the 12 key active ingredients that differed significantly among the three camellias, catechin, galloylcatechin, and epicatechin were the most prominent, highlighting the central role of catechins in the predicted anti-inflammatory, antioxidant, antibacterial, and antiviral activities. Other important compounds, including luteolin 3′-methyl ether 7-malonylglucoside, pelargonidin-3-O-β-D-glucoside, and apigenin 7-(6″-ethylglucuronide) were also identified and consistent with previously reported findings, reinforcing their potential as bioactive ingredients in the genus *Camellia*.

### 3.3. Unique Bioactive Floavonoids in Genus Camellia May Play Important Role

An increasing number of unique, bioactive flavonoids have been identified in the genus *Camellia*. Notable examples include camelliagenins A, B, and C [44]; camelliasaponins B1, B2, C1, and C2 [45], camellianoside [46], 2,3-digalloyl-O-α-D-glucopyranoside and 2,3-digalloyl-O-β-D-glucopyranoside [43]; and camelliatannin I [47] all isolated from *C. japonica*. Additionally, sanchakasaponins C and D, found in *Camellia multipetala*, *Camellia petelotii* var. grandiflora, and *Camellia longzhouensis*, have been identified as key bioactive compounds responsible for senescent cell clearance [38]. Two new flavonol polysaccharides, named hirsutaosides A and B, were isolated from the leaves of *Camellia hirsuta*, a native species of golden camellias native to Vietnam, and shown to possess anti-diabetic properties [48]. In the present study, camelliagenin A was detected in both XZ and SC samples; however, due to its low confidence level (Level 3), it was not discussed further here. Future studies will aim to validate this compound through standard comparisons or quantitative analyses. In addition, izalpinin was detected in XZ but not in LX or SC. Originally isolated from *Alpinia oxyphylla*, izalpinin has been implicated in the treatment of diseases such as Alzheimer’s disease [34]. while, its role within camellias is unverified and should be tested experimentally in the upcoming studies. These findings suggest that izalpinin, camelliagenins, and other unique or specific compounds derived from camellias warrant further study to elucidate their therapeutic potential, while its role within camellias is unverified, and should be tested experimentally in future work.

## 4. Materials and Methods

### 4.1. Materials

#### 4.1.1. Preparation of Flowers

Two promising camellias were selected for comparison with a commonly used reference camellia. These included *Camellia japonica* (SC), widely applied in cosmetic and medicinal research; *Camellia* ‘High Fragrance’ (LX), which already has a certain degree of industrial application; and *C. japonica* ‘Kōshi’ (XZ), known for its distinctive fragrance.

Fresh flowers from SC, LX, and XZ were collected during the winter of 2023–2024. Samples were randomly divided into six biological replicates, freeze-dried, and stored with silica gel at room temperature (20–25 °C) until extraction and analysis.

#### 4.1.2. Preparation of Flower Extracts

Extraction of camellia metabolites was performed following previously published methods with minor modifications [11,49]. Briefly, 60 mg of powdered flower tissue was transferred into a 1.5 mL Eppendorf tube, to which 700 μL of ice-cold methanol–water (1:4, *v*/*v*) containing 4 μg/mL L-2-chlorophenylalanine was added. Samples were pre-cooled at −40 °C for 2 min and homogenized using a grinder (60 Hz, 2 min). The homogenates were subjected to ultrasonic extraction for 30 min in an ice–water bath, followed by incubation at −40 °C for 2 h. Extracts were centrifuged at 13,000 rpm for 10 min at 4 °C, and 150 μL of the resulting supernatant was collected from each sample using crystal syringes. Supernatants were filtered through 0.22 μm microfilters and transferred into liquid chromatography (LC) vials, which were stored at −80 °C until liquid chromatography–mass spectrometry (LC–MS) analysis. Quality control (QC) samples were prepared by pooling aliquots from all extracts to generate a representative mixture.

### 4.2. Metabolomics Analysis

#### 4.2.1. Condition for Metabolomics Analysis

Non-targeted metabolite profiling of camellia extracts was conducted according to previously described methods with slight modifications [50]. Analyses were performed on an ACQUITY UPLC I-Class Plus system (Waters Corporation, Milford, CT, USA) coupled with a Q-Exactive mass spectrometer equipped with a heated electrospray ionization (HESI) source (Thermo Fisher Scientific, Waltham, MA, USA). Data were acquired in both positive and negative ion modes. Chromatographic separation was achieved using an ACQUITY UPLC HSS T3 column (1.8 μm, 2.1 × 100 mm; Waters Corp.). The mobile phases consisted of (A) water with 0.1% formic acid (*v*/*v*) and (B) acetonitrile with 0.1% formic acid (*v*/*v*). The gradient program was as follows: 0.01 min, 5% B; 2 min, 5% B; 4 min, 30% B; 8 min, 50% B; 10 min, 80% B; 14 min, 100% B; 15 min, 100% B; 15.1 min, 5% B; and 16 min, 5% B. The flow rate was 0.35 mL/min, and the column temperature was maintained at 45 °C. All samples were kept at 10 °C during analysis, and the injection volume was 3 μL.

The mass spectrometer was operated over a mass-to-charge (*m*/*z*) range of 70–1050. The resolution was set to 60,000 for full MS scans and 15,000 for higher-energy collisional dissociation tandem MS (HCD-MS/MS) scans. Collision energies were 10, 20, and 40 eV. The additional instrument parameters were as follows: spray voltage, 3800 V (+) and 3200 V (−); sheath gas flow rate, 35 arbitrary units; auxiliary gas flow rate, 8 arbitrary units; capillary temperature, 320 °C; auxiliary gas heater temperature, 350 °C; and S-lens RF level, 50.

#### 4.2.2. Metabolite Identification

Raw LC-MS data were processed using Progenesis QI V2.3 software (Nonlinear Dynamics, Newcastle, UK) for baseline filtering, peak identification, integration, retention time correction, peak alignment, and normalization. The main processing parameters were set to a precursor tolerance of 5 ppm, product tolerance of 10 ppm, and a production threshold of 5%. Compound identification was based on accurate mass-to-charge ratio (*m*/*z*), secondary fragmentation patterns, and isotopic distribution. Databases used for identification included the Human Metabolome Database (HMDB; https://www.hmdb.ca/, (accessed on 2 December 2024)), the metabolite and tandem MS database (METLIN; http://metlin.scripps.edu/, (accessed on 2 December 2024)), LipidMaps (https://www.lipidmaps.org/, (accessed on 2 December 2024)), and in-house databases developed by Shanghai Luming Biological Technology Co., LTD (Shanghai, China).

#### 4.2.3. Data Analysis

Extracted data were processed by removing peaks with missing values (ion intensity = 0) in more than 50% of samples. Zero values were replaced with half of the minimum observed value. Compounds were screened based on qualitative confidence scores, and those scoring below 36 (out of 80) were excluded as unreliable. The remaining metabolites were categorized into four confidence levels according to retention time (RT) and fragmentation score. Level 1 metabolites (RT: ±0.3 min (18 s) and fragmentation score ≥ 45) were selected for further analysis.

A combined data matrix, integrating both positive and negative ion mode data, was constructed and imported into R for statistical analyses. Principal component analysis (PCA) was applied to assess sample distribution and the overall stability of the analytical process. Orthogonal partial least squares-discriminant analysis (OPLS-DA) and partial least squares-discriminant analysis (PLS-DA) were utilized to distinguish metabolites differing among groups. To prevent overfitting, we applied 7-fold cross-validation and 200 response permutation tests (RPT) to evaluate the quality of the model. Variable importance in projection (VIP) values obtained from the OPLS-DA model were used to rank metabolites based on their contribution to group discrimination. Statistical significance was assessed with two-tailed Student’s *t*-tests. Indicators (FC, VIP and *p* value) for initial and deep analysis were set according to previously report [51] with the difference in FC value. FC ≥ 8 or ≤1/8 and *p* values < 0.05 were set as the screening criteria for metabolites according to the quantity and quality of identified metabolites. Significantly differential metabolites were further selected for deep analysis using the defend criteria (VIP > 1.0, FC value. FC ≥ 8 or ≤1/8, and *p* values < 0.05). All graphical representations were generated using Origin Pro 9.0 (OriginLab Corporation, Northampton, MA, USA).

### 4.3. Network Pharmacology Analysis

Network pharmacology analysis was performed based on reports on camellias [15,16], with minor modifications. Gene Cards (https://www.genecards.org/, (accessed on 15 May 2025)) was used as the primary disease-related database, while STRING (https://cn.string-db.org/, (accessed on 15 May 2025)) and Metascape (https://metascape.org/, (accessed on 15 May 2025)) served as platforms for network pharmacology analysis. The procedures are described below.

#### 4.3.1. Screening of Cross-Targets Between Ingredient-Related Targets and Disease-Associated Targets

Potential targets related to active ingredients were predicted using Swiss Target Prediction (http://www.swisstargetprediction.ch/, (accessed on 20 May 2025)) with the condition Probability > 0, restricted to *Homo sapiens*. A total of 71 phenylpropanoids and polyketides were submitted, of which 67 were identified and retained for further analysis. The UniProt database (https://www.uniprot.org/, (accessed on 20 May 2025)) was then used to standardize the relevant targets. Targets related to the selected camellia ingredients were obtained by merging and removing duplicates in an Excel spreadsheet.

Disease-associated targets were obtained from the Gene Cards database, which provides comprehensive gene-specific information and interactive pathway maps. The keywords “inflammation”, “oxidative stress”, “antibacterial”, and “antiviral” were queried separately. The retrieved targets were standardized through UniProt, and duplicates were removed. The resulting target sets represented the disease-related pools for anti-inflammatory, antioxidant, antibacterial, and antiviral activities.

To identify overlapping targets, Venn analysis was performed using the Chinese Version of Bioinformatics (https://www.bioinformatics.com.cn/, (accessed on 28 May 2025)). This yielded the intersecting targets between phenylpropanoids/polyketides and the four therapeutic functions.

#### 4.3.2. Protein–Protein Interaction (PPI) Construction and Core Target Selection

The STRING database was used to construct protein–protein interaction (PPI) networks for the intersecting targets, with the species limited to *Homo sapiens* and the confidence score set to 0.900. Network visualization and topological analysis were performed using Cytoscape 3.10.2 to identify core targets.

#### 4.3.3. Gene Ontology (GO) and Kyoto Encyclopedia of Genes and Genomes (KEGG) Enrichment Analysis

Functional enrichment analysis of intersecting targets was carried out using the Metascape platform [14]. Both Gene Ontology (GO) terms and Kyoto Encyclopedia of Genes and Genomes (KEGG) pathways were analyzed, with the species set to *Homo sapiens* and the significance threshold set at *p* < 0.01. The results were visualized using the Chinese Version of Bioinformatics (https://www.bioinformatics.com.cn/, (accessed on 28 May 2025)), highlighting the top 10 GO terms and the top 20 KEGG pathways.

#### 4.3.4. Network Diagram Construction and Key Ingredient Identification

We constructed a key plant–component–target–pathway network for camellias by integrating the active ingredients identified from the three species, their intersection targets with disease-related proteins, and the top 20 KEGG signaling pathways. The network was constructed using Cytoscape 3.10.2 software. Network Analyzer was further employed to perform topological analysis on the network. Degree centrality is widely used to measure node importance and connectivity in network pharmacology analysis and camellia was included [17]. We used degree as the criterion to measure the importance of phenylpropanoid and polyketide ingredients on four studied diseases, degree was calculated based on the network diagram constructed using Cytoscape 3.10.2.

## 5. Conclusions

This study indicated the diversity of metabolites in the Genus *Camellia.* Among the 71 significantly different phenylpropanoids and polyketides identified, 54 were common across all three camellias, while 17 were unique, potentially reflecting genetic variation or diversity. There are approximately 30,000 camellia species and cultivars in total, which opens up the perspective of discovering new resources, such as those in the sections *Chrysantha*, *Camellia*, *Thea*, etc. Network pharmacology analysis further identified several key active phenylpropanoids and polyketides associated with anti-inflammatory, antioxidant, antibacterial, and antiviral activities. These compounds, including flavonoid glycosides, flavans, biflavonoids, polyflavonoids, and a flavone, may serve as valuable resources for developing applications in cosmetics, food, and medicine, as well as for breeding new camellia varieties with enhanced functional properties. Future research will be launched to validate the predicted key active compounds through standard comparisons or quantitative analyses, test their functions experimentally, and discover more superior camellia resources to meet human needs.

## Figures and Tables

**Table 1:** Compounds with the top 10 degree scores in the network and their accumulation levels across the three cultivars.

Metabolites	Formula	PubChem ID	Sub Class	Degree	Accumulation Level Across the Three Cultivars ^1^		
					Average (XZ)	Average (LX)	Average (SC)
Izalpinin	C16H12O5	5318691	Flavones	28	16.09	7.23	7.23
Isokaempferide	C21H22O10	42607834	Flavonoid glycosides	26	14.87	21.85	12.35
pelargonidin-3-O-beta-D-glucoside	C21H20O10	443649	Flavonoid glycosides	18	9.51	15.92	10.44
Catechin 5,3′-di-O-gallate	C29H22O14	15689619	Flavans	17	18.04	16.12	11.66
2′,4′,6′,beta-Tetrahydroxychalcone 2′-glucoside	C21H22O10	42607637	Flavonoid glycosides	16	15.57	10.55	7.23
7-Galloylcatechin	C22H18O10	74490567	Flavans	16	18.47	12.94	15.40
Pinocembroside	C21H22O9	46881227	Flavonoid glycosides	14	17.49	11.54	8.17
ent-Epicatechin(4alpha->8)catechin	C30H26O12	130556	Biflavonoids and polyflavonoids	14	16.16	7.23	9.02
Apigenin 7-(6″-ethylglucuronide)	C23H22O11	14309759	Flavonoid glycosides	13	18.07	8.66	7.23
Luteolin 3′-methyl ether 7-malonylglucoside	C25H24O14	131752190	Flavonoid glycosides	12	7.48	14.92	7.23
8-Glucopyranosylprocyanidin B2	C36H36O17	21637579	Biflavonoids and polyflavonoids	12	17.65	20.26	12.70
Isosakuranetin 7-alpha-L-arabinofuranosyl-(1->6)-glucoside	C27H32O14	42607954	Flavonoid glycosides	12	15.81	19.16	8.00

^1^ The accumulation level shown in the table represent the relative expression content of each compound.

## Data Availability

Data are contained within the article and Supplementary Materials.

## Supplementary Materials

The following supporting information can be downloaded at https://www.mdpi.com/article/10.3390/plants14192967/s1, Figure S1: A presentation of chance permutation at 200 times used for the discrimination among three camellias. Table S1: Phenylpropanoid and polyketide for network pharmacology.

## Abbreviations

The following abbreviations are used in this manuscript:

XZ	*Camellia japonica* ‘Kōshi’
LX	*Camellia* ‘High Fragrance’
SC	*Camellia japonica*
LC	Liquid Chromatograph
MS	Mass Spectrometer
ESI	Electrospray ionization
PCA	Principle Component Analysis
OPLS-DA	Orthogonal Partial Least-Squares–Discriminant Analysis
PLS-DA	Partial Least-Squares–Discriminant Analysis
VIP	Variable importance in projection
PPI	Protein–protein interaction
GO	Gene Ontology
KEGG	Kyoto Encyclopedia of Genes and Genomes
AGE-RAGE	Advanced glycation end product—(receptor for AGEs)
