# Supplementary figures and images for: Non-Targeted Metabolomics and Network Pharmacology Reveal Bioactive Metabolites and the Medicinal Potential of Three Ornamental Camellia Flowers
Source: Plants (Basel). 2025 Sep 24;14(19):2967. doi: 10.3390/plants14192967 (PMC12525878; doi:10.3390/plants14192967)

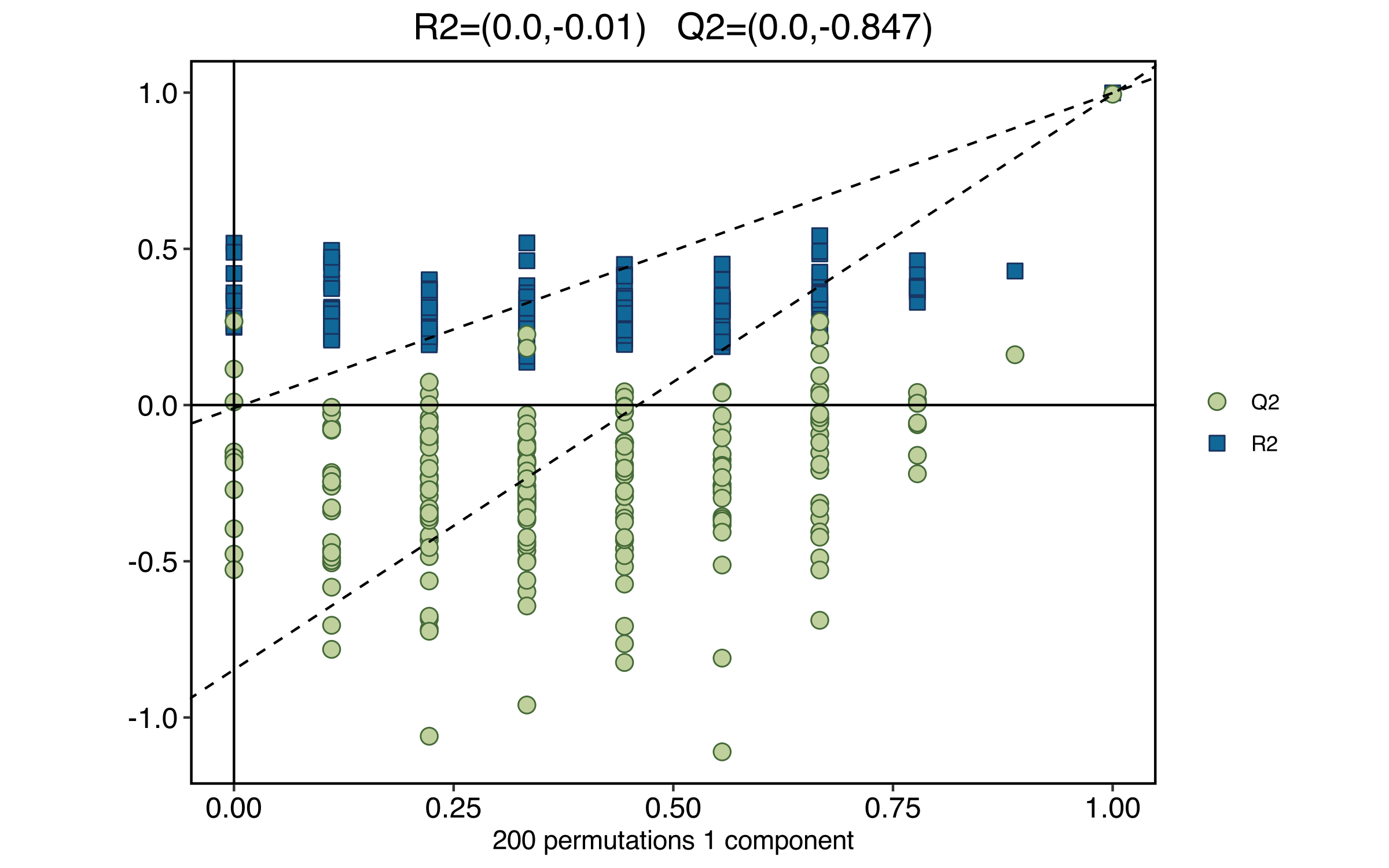

Supplement: Supplementary file 1 [file plants-14-02967-s001.zip › Supplementary materials/Figure S1(permutation-XZ-vs-LX-vs-SC).png]
